# Squamous cell carcinomas in linear epidermal naevi

**DOI:** 10.1111/ced.13704

**Published:** 2018-06-28

**Authors:** A. Dubois, S. Rannan‐Eliya, A. Husain, N. Rajan, T. Oliphant

**Affiliations:** ^1^ Department of Dermatology Royal Victoria Infirmary Newcastle upon Tyne UK; ^2^ Department of Plastic Surgery Royal Victoria Infirmary Newcastle upon Tyne UK; ^3^ Department of Cellular Pathology Royal Victoria Infirmary Newcastle upon Tyne UK; ^4^ Institute of Genetic Medicine University of Newcastle upon Tyne Newcastle upon Tyne NE1 3BZ UK

Linear keratinocytic epidermal naevi (KEN) within the lines of Blaschko are thought to arise as a result of postzygotic mutations in genes that influence epidermal homeostasis, including *FGFR3* 1 and *HRAS*.^2^ Squamous cell carcinoma (SCC) arising in epidermal naevi is rare; however, the recurrent development of multiple SCCs arising in KEN rather than normal skin suggests that some epidermal naevi predispose to SCC formation. We report a patient with two SCCs arising in a linear KEN, and review the existing literature.

**Figure 1:**
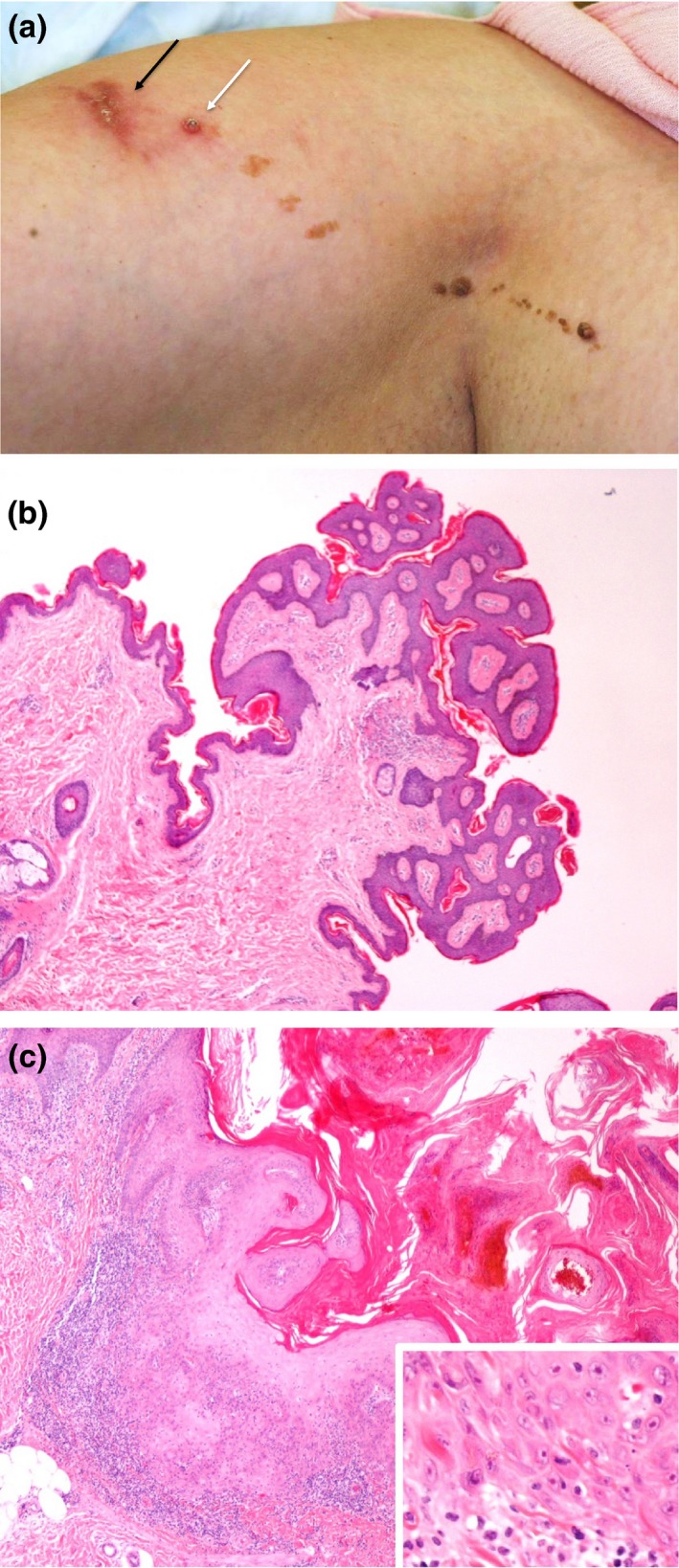
(a) Linear epidermal naevus extending from the right upper arm to right axilla. Black arrow indicates site of the first squamous cell carcinoma (SCC), white arrow indicates second SCC. (b) Keratinocytic epidermal naevus showing thickened epithelium with papillomatosis, increased basal hyperpigmentation and an acanthotic epidermis; (c) SCC presenting as a crateriform lesion with marked eosinophilia of epidermis with (inset) well‐differentiated squamous cells with nuclear cytological abnormalities, pushing into the dermis. Haematoxylin and eosin, original magnification (b) × 20; inset × 40.

A 51‐year‐old woman presented with a growing, bleeding nodule arising in a long‐standing KEN on her right upper arm (Fig. 1a). She had Fitzpatrick skin type II and no history of excess ultraviolet light exposure.

Physical examination revealed a linear warty pigmented naevus extending from the axilla down the right upper inner arm. A pink nodule measuring 15 × 10 mm with overlying haemorrhagic crust was noted within the naevus. An excision biopsy showed a well‐differentiated SCC with a thickness of 2.1 mm arising on a background of a papillomatous and hyperkeratotic epidermis, consistent with an epidermal naevus (Fig. 1b). Four months later, the patient developed a second papule, 5 × 5 mm in size, in a different area of the naevus. Excision of this lesion confirmed a second primary SCC, which was moderately differentiated with a thickness of 1.8 mm (Fig. 1c). After her second SCC, our patient opted for complete excision of her KEN.

Epidermal naevi arise from the pluripotent cells of the embryonic ectoderm, due to somatic mosaicism, and are classically seen as circumscribed lesions in a blaschkoid pattern.^3^ Benign and asymptomatic, they are not usually thought of as a cause for clinical concern. There are, however, 10 previously reported cases of SCC and 5 of keratoacanthoma (KA) developing within KEN (Table 1). The mean age of those affected was 45 years (range 17–82 years), and there was no sex preponderance. All cases of SCC describe a single lesion, except for one case in which two SCCs developed 4 months apart, a similar interval to our case. Multiple lesions were seen in two of the five patients with KA. Of the 15 cases, 4 had poorly differentiated lesions histologically, and all of these metastasized: two at the time of excision, one 6 weeks later and one 8 months later. In one of these four cases, the patient died as a result of metastatic SCC.

**Table 1:** Reported cases of cutaneous squamous cell carcinoma and keratoacanthoma arising in linear keratinocytic epidermal naevi

Patient	Age, years*	Sex	Ethnicity	Clinical presentation	Histology
1	17	Female	White Caucasian	Nodule on right breast arising in a linear epidermal naevus†	Well‐differentiated SCC
2	26	Female	NR	2 dome‐shaped nodules 1 cm in size, appearing suddenly on a long‐standing linear epidermal naevus on right upper limb	KA
3	32	Male	White Caucasian	3 keratotic dome‐shaped nodules arising in the distal aspect of a longstanding epidermal naevus on left upper limb	Two KAs
4	69	Male	Israeli	2 cm ulcer arising on a verrucous plaque on right chest. Subsequent metastases to lymph nodes and later to lungs	Metastatic, poorly differentiated SCC
5	74	Male	Japanese	Widespread warty naevus since birth; 13 × 15 cm ulcerated nodule on middle back	SCC
6	23	Female	Chinese	Nodule arising in epidermal naevus behind right ear	KA
7	27	Female	NR	Raised nodule on a background of linear papules on right thigh†	SCC
8	28	Female	NR	Two SCCs arising in a linear epidermal naevus on the right upper arm	Both tumours were well‐differentiated SCCs
9	81	Female	African‐American	Left thigh: 3‐year history of a single nodule, 40 × 25 cm at same site as a previously noted epidermal naevus	Well‐differentiated SCC
10	82	Female	Japanese	25 mm tumour on a 45 × 40 mm plaque in right axilla. Metastasis to local LNs detected at excision	Well‐differentiated SCC with LN metastasis
11	28	Female	White Caucasian	10 cm tumour on left labium extending to anus with regional lymphadenopathy arising on a congenital verrucous epidermal naevus	Invasive SCC
12	50	Male	Asian	3 cm nodule on back within localized verrucous naevus. Fatal metastatic cutaneous SCC evolving from a localized verrucous epidermal naevus†	Poorly differentiated SCC
13	56	Male	Indian	Asymptomatic, rapidly growing nodule on a linear epidermal naevus on the neck	KA
14	37	Male	NR	4 × 3 cm SCC on right thigh arising in a verrucous epidermal naevus	Well‐differentiated SCC
15	47	Male	Indian	Asymptomatic growing nodule arising in an epidermal naevus on the forehead	KA

KA, keratoacanthoma; LN, lymph node; NR, not recorded; SCC, squamous cell carcinoma.*At presentation; †all naevi were documented to be apparent at birth except those marked (†), for which this information was not specifically detailed. Table references are available at: Open Science Framework: https://osf.io/t6pwh/ (Rajan N. Squamous cell carcinomas in linear epidermal naevi, 2018).

Our case highlights the importance of recognizing that SCC can arise in longstanding KEN, and may metastasize. Knowing which patients may be at risk is challenging, as there are no obvious clinical markers that highlight predisposed individuals. Future research may highlight specific genetic mutations in a subgroup of KEN that is associated with increased SCC development. The knowledge that SCC can occur occasionally and recurrently in KEN should inform and influence clinical decision‐making regarding monitoring or pre‐emptive interventions. Patients should be advised to report growing nodules for clinical evaluation presenting after childhood. Excision of these naevi, if surgically feasible and aesthetically acceptable, may be a prudent option for patients who develop SCC.
